# Impacts of propolis and *Spirulina platensis* supplementation on growth, nutrient digestibility, and gut microbiota of Japanese quails under heat stress

**DOI:** 10.1038/s41598-025-17082-2

**Published:** 2025-08-28

**Authors:** Mohamed A. Fawaz, Mahmoud A. Ali, Rafat Khalaphallah, Hamdy A. Hassan, Abdalla H. H. Ali

**Affiliations:** 1 Department of Animal and Poultry Production, Faculty of Agriculture, South Valley University, Qena, 83523 Egypt; 2 Department of Plant Protection, Faculty of Agriculture, South Valley University, Qena, 83523 Egypt; 3 Department of Agricultural Botany (Microbiology), Faculty of Agriculture, South Valley University, Qena, 83523 Egypt

**Keywords:** Propolis, *Spirulina platensis*, Quails, Performance, Nutrient digestibility, Heat stress, Animal physiology, Climate-change impacts

## Abstract

This experiment determined the effects of Propolis (PRO) and *Spirulina platensis* (SP) powder on Japanese quail growth performance, nutrient digestibility, and certain serum parameters under heat stress condition. A total of two hundred unsexed Japanese quails (seven days old) were randomly assigned to four treatments. Each treatment included five replicates (10 quails per each). For five weeks, quail chicks were fed basal diet without supplementation (control), quail chicks were fed basal diet supplemented with 400 mg/kg of PRO, quail chicks were fed basal diet supplemented with 1 g/kg of *Spirulina platensis* powder (SP), and quail chicks were fed basal diet supplemented with same amount of propolis and *Spirulina platensis* powder (Mix). Data analysis revealed that supplementation of PRO at 400 mg/kg to quails diet greatly improved (*P* = 0.001) body weight, body weight gain, feed conversion ratio (FCR) as well as improved ether extract, crude protein digestibility (*P* = 0.028), serum concentration of albumin, serum total cholesterol in addition to reduced (*P* < 0.001) the count of *Bacillus SPP*, *Escherichia coli (E. coli), Clostridium Spp, and Enterococcus Spp* under heat stress condition. Dietary inclusion of SP at a dose of 1 g/kg alone or in combination with PRO significantly (*P* = 0.001) improved FCR, serum concentration of albumin in addition to reduced digestibility of ether extract and serum concentration of triglyceride (*P* = 0.001) compared to the control group. In conclusion, when used individually, propolis (400 mg/kg) and Spirulina (1 g/kg) improved performance and health status of Japanese quails under heat stress; however, their combination did not yield additive benefits.

## Introduction

One of the most common environmental stressors that have a detrimental impact on the welfare, health, and productivity of commercial poultry in Upper Egypt is high ambient temperature during the length of the summer season. Reduced immune function, elevated free radical generation, and lipid peroxidation of cell membranes are some of the significant biochemical and physiological effects of heat stress^1^. High ambient temperatures, a typical environmental stressor, increase the need for vital nutrients and antioxidants in bird’s diets to reverse the declines in the health and performance of birds, particularly in highly productive systems^2,3^. Many studies are interested in nutritional changes to lessen the detrimental effects of heat stress on poultry because effective cooling of poultry housing is expensive. Nutritional supplements of electrolytes and vitamins^4,5^, probiotics^6^, prebiotics^7^, organic acids and phytogenics^8,9^, propolis^10^ and other feed additives or management techniques^11^ have been shown in numerous studies to help mitigate the negative effects of heat stress on poultry performance.

Propolis is a naturally occurring resinous compound that bees collect from plant buds and exudates and combine with wax and enzymes. Flavonoids are a significant component of plant-produced propolis which can potentially improve chickens efficiency through enhanced gut health, nutritional digestion, and absorption^12^. The composition of propolis can vary depending on the hive, locale/geographic source, and season^13,14^. The pigmentation of propolis is also affected by the availability of resources around the hive^15^. The chemical composition of propolis varies slightly depending on the source of collecting. Propolis contains 7% bee wax, 5% bee pollen, 55% resin-polyphenolic fraction, 30% aromatic essential oils, and 3% other minor components such as vitamins (A, D, C, E, and B1, B2, and B6), folate, and niacin, as well as some macro and micro minerals such as magnesium, calcium, manganese, copper, zinc, nickel, and vanadium^16–18^. Because propolis includes bioactive components in addition to a high concentration of vitamins and minerals, these compounds are assumed to contribute to its pharmacological, biological, and potential^19^. Also, among the most bioactive chemical components of propolis are flavonoids and phenolic acids, and they are principally responsible for its potent bactericidal, antifungal, and antioxidant effects^19,20^. As a result, propolis has the potential to boost quails immunity, health, and production.

Algae are a source of critical biologically valuable components, making sourcing these chemicals from natural habitats a possible technique for developing creative diets^21,22^. Spirulina (*Arthrospira platensis***)** is a cyanobacterium, and it is one of the oldest algae species on our planet, with origins reaching back nearly 3 billion years^23^. Spirulina (Sp) contains high levels of protein, phenolic acids, and micronutrients, including potassium, calcium, magnesium, iron, zinc, provitamin β-carotene (vitamin A), thiamine (vitamin B1), riboflavin (vitamin B2), nicotinamide (vitamins B3), pyridoxine (vitamins B6), folic acid (vitamins B9), cyanocobalamin (vitamin B12), vitamin C, vitamin D, and vitamin E^24,25^. Microalgae have high nutrient digestibility, comparable to other vegetable diets and feeds^26,27^. Spirulina has the ability to partially substitute traditional protein sources, including soybean meal^28^. According to the American Food and Drug Administration (FDA) and the European Food Safety Authority the *Spirulina platensis* has been classified safe^29^. Spirulina may provide therapeutic benefits, boost intestinal lactobacilli, reduce nephrotoxicity from drugs and heavy metals, and protect against radiation^30,31^. It also improves immune function, reproduction, and growth^32^. Strong anti-inflammatory and antioxidant properties are exhibited by C-phycocyanin, an essential component of Sp^33^. Supplementing diets with microalgae can improve growth rate, livability, feed utilization, and carcass quality. Zaghari and Hajati^34^ recommended supplementing Japanese quails with high levels of *Spirulina platensis* to improve their immune responses and growth performance.

The combination of propolis and *Spirulina platensis* presents a promising synergistic approach due to their complementary bioactive properties. While each has demonstrated individual therapeutic benefits, recent interest has focused on the enhanced efficacy that may result from their combined use. This combination could potentially yield additive or synergistic effects in modulating oxidative stress, enhancing physiological and nutritional responses or improving overall health outcomes of Japanese quails. Therefore, evaluating the combined application of propolis and spirulina platensis is scientifically justified and may offer novel insights into natural compound-based therapies. Therefore, the objective of this study was to evaluate the impacts of propolis and *Spirulina platensis* powder on performance, carcass criteria, nutrient digestibility, cecal microbiota and some serum parameter of growing Japanese quails under heat stress conditions.

## Materials and methods

### Ethical statement

The current experiment was Approved by the Ethics Committee of the Local Experimental Animals Care Committee of Department of Animal and Poultry production, Faculty of Agriculture, South Valley University, Egypt (Approval Code: 01/01/12/24). All experimental procedures involving animals were carried out in strict accordance with the relevant institutional guidelines and regulations, and following ARRIVE guidelines.

### Experimental animals and design, and feed preparation

A total of two hundred unsexed Japanese quails (Seven days old) were randomly assigned to four treatments. Each treatment included five replicates (10 quails per each) were procured from a hatchery of the Experimental Poultry Farm, Department of Animal and Poultry Production, Faculty of Agriculture, South Valley University, Qena, Egypt. The quails were housed in a standard cage (100 × 40 × 40 cm) with a light cycle of 24 h. During the five-week experiment, both feed and water were freely available to the chickens during the experiment. Birds were fed in mash form in accordance with their treatment. According to NRC^35^, the basal diet was formulated and prepared in order to satisfy the nutrient requirements (Table 1). For 5 weeks, quail chicks were fed basal diets without supplementation (control), quail chicks were fed basal diets supplementation with 400 mg/kg of propolis (PRO), quail chicks were fed basal diets supplemented with 1 g/kg of *Spirulina platensis* powder (SP), and quail chicks were fed basal diets supplemented with same amount of propolis and *Spirulina platensis* powder (Mix).

**Table 1:** Composition and chemical analysis of the basal diet.

Ingredients (g/kg)	Starter diet
Sorghum	255
Maize	275
Soybean meal (44% CP)	300
corn gluten meal (60% CP)	102.4
Vit & Min. Premixa	3.02
Corn oil	30
Dicalcium phosphate	20
Limestone	10
Salt	3.04
DL-methionine	0.42
L- lysine HCl	1
Total	1000
Nutrient Analysis (g/kg)	
Dry matter	91.5
Crude protein	241.4
Ether extract	52.2
Crude fibre	25.24
Ash	63.2
Ca (g/kg)	12.2
Available phosphorus	7.7
ME, (Kcal/kg diet)	3000

Supplied vitamin-mineral premix contains per kg of diet: 2400.000 IU vitamin A; 1000.000 IU vitamin D; 800 mg vitamin K;16.000 IU vitamin E; 650 mg vitamin B1; 1.600 mg vitamin B2; 1.000 mg vitamin B6; 6 mg vitamin B12; 8.000 mg niacin; 400 mg folic acid; 3.000 mg pantothenic acid; 40 mg biotin; 3.000 mg antioxidant; 80 mg cobalt; 2.000 mg copper; 400 mg iodine; 1.200 mg iron; 18.000 mg manganese; 60 mg selenium; 14.000 mg zinc.

### Conditions of the experimental environment

The current investigation was conducted during the length of the summer season. Throughout the study, daily values of minimum and maximum room temperatures, and the humidity %, were recorded and averaged weekly. Consequently, the equivalent temperature-humidity index was determined. Heat stress was graded as absent (THI < 27.8), moderate (THI = 27.8–28.8), severe (THI = 28.9–29.9), or very severe (THI > 30.0)^36,37^. THI was calculated according the following formula:

$${\text{THI}} = {\text{Tdb}}{-}\left[ {\left( {0.31{-}0.31{\text{RH}}} \right)*\left( {{\text{Tdb}} - 14.4} \right)} \right].$$

### Preparation of* Spirulina platensis*

The Department of Agricultural Botany (Microbiology), Faculty of Agriculture, South Valley University, 83523 Qena, Egypt, is where the *Spirulina platensis* algae were obtained. *Spirulina platensis* (SP) was cultivated in normal synthetic Zarrouk medium (Zarrouk^38^). at 35 °C ± 2 with constant illumination (2000 l×). Primal filtration was used to extract the algal biomass from the culture media following a one-month incubation period. After being freeze-dried, the algae were ground into a fine powder. A dry matter basis, SP’s chemical composition was as follows: total ash 33.71%, crude protein 45.43%, moisture 5.58%, ether extract 8.24%, and crude fiber 0.95%.

### Collection and preparation of propolis

Propolis was collected from active beehives located near Acacia trees in Qena, Egypt. Acacia trees were specifically chosen due to their dominance in the region and their known impact on the chemical properties of propolis. The collection was conducted during the spring season of 2024, a period optimal for resin production. Propolis samples were manually scraped from the hive walls and frames using sterile tools to prevent contamination.

#### Processing of raw material of propolis

The collected raw propolis samples were cleaned by removing visible debris, such as wood particles, beeswax, and other impurities, using sterilized tweezers.

The cleaned propolis was broken into smaller pieces using a sterile mortar and pestle. This step was performed under controlled conditions to prevent contamination.

#### Extraction method of propolis

A 70% ethanol solution was prepared, as it is widely recognized for its ability to extract bioactive compounds such as flavonoids and phenolic acids from propolis. The crushed propolis was weighed, and a 1:10 ratio of propolis to ethanol (w/v) was maintained. For instance, 100 g of propolis was added to 1 L of ethanol. The mixture was stirred continuously using a magnetic stirrer for 48 h at room temperature (22–25 °C) to ensure thorough extraction. After stirring, the mixture was filtered through Whatman No. 1 filter paper to separate the ethanolic extract from solid residues^39^.

#### Concentration and Drying of Propolis

The filtered ethanolic extract was concentrated under reduced pressure using a rotary evaporator set at 40 °C to remove most of the ethanol, yielding a semi-solid extract. The concentrated extract was further dried in a sterile air-drying cabinet to ensure the complete removal of residual ethanol, resulting in a fine propolis powder. The dried powder was stored in amber-colored airtight glass containers at 4 °C to maintain stability and protect the bioactive compounds from light and humidity.

The chemical constitution of the same source of propolis powder employed in the present study was already described by Mowafi et al.^40^, reporting the existence of various biologically active substances. The main components detected were pinocembrin, galangin, and chrysin (6.45, 4.52, and 3.01% respectively), together with smaller amounts of kaempferol (1.53%), quercetin (1.33%), caffeic acid (1.47%), ferulic acid (1.46%), and p-coumaric acid (1.01%).

### Productive performance parameters

The body weights of quail chicks in each replicates were recorded on days 1, 10, 20, 30, and 42 of age. To determine how much feed had been consumed in each replicates, feed residue was measured on the day when the birds were weighed. Throughout the experiment, mortality was monitored daily. Feed conversion ratio (FCR) was calculated by the following formula:

$$\text{Feed conversion ratio }(\text{FCR})=\frac{\text{Daily feed intake }}{\text{ Daily body weight gain}}$$

### Nutrient digestibility trial

At the end of the experiment, for the last five days of the experiment, 40 quail chicks in all, (two per replicate), were kept in metabolic cages made for separate housing. This made it possible to accurately collect all of the excreta from each bird separately. The birds were fasted for 12 h before and after subjected to a five days experiment. Using trays placed underneath the cages, the excreta from each broiler was collected every day for a continuous five days. Only genuine excreta were kept for analysis after all feathers and feed particles were meticulously removed by hand using forceps to guarantee sample accuracy. The total amount of excreta from each replicates were collected every day and weighed and feed intake was recorded. All excreta were frozen at − 20 °C until it was ready for chemical analysis. Prior to chemical examination, the excreta were homogenised. Feed and excreta were further dried in an oven before being processed into a fine powder using a centrifugal mill and a 1 mm screen. According to AOAC^41^, diets and excreta samples were evaluated for dry matter (930.15), ash (942.05), and ether extract (954.02). The gross energy was measured using a Parr adiabatic bomb calorimeter (Moline, IL, USA). The nutrient digestibility was determined using the following formula:

$$\text{Nutrient digestibility},\text{ \% }=\frac{(\text{Nutrient in feed}-\text{Nutrient excreted in feces})}{\text{Nutrient in feed }} \times 100$$

### Carcass criteria

Quails aged 35 days were starved overnight although had access to water. 10 birds were randomly selected for each treatment (two/replicate), weighted, slaughtered, and plucked by the Islamic method. After removing the head, neck, shanks, viscera, digestive tract, spleen, liver, gizzard, heart, and abdominal fat, the dressing weight was determined by weighing the rest of the body. Each quail chicks liver, heart, spleen, empty gizzard, abdominal fat, and cecum were weighed and expressed as a proportion of its live body weight. The dressing % was estimated using the following formula:

$$\text{Dressing percentage \%}=\frac{\text{ carcass weight }}{\text{Live body weight }} \times 100$$

### Blood collection and laboratory analysis

A total of 10 quail chicks from each treatment were randomly selected to represent the pen at the end of the experiment. To collect serum, blood was drawn from the wing vein with sterile needles and syringes in vacutainer tubes. Before blood was extracted, no feed was removed from the feeder. Following natural separation, the serum was centrifuged at room temperature for 10 min (4000 RPM). Serum was collected in tubes and stored at − 20 °C for further analysis. Serum total protein (Catalog No.: ZL-310 001); albumin, (Catalog No.: ZL-211 001), glucose (Catalog No.: ZL-250 001), total cholesterol (Catalog No.: 230 001), triglycerides (Catalog No, 314 010), as well as urea (Catalog No.: ZL-320 001) and creatinine (Catalog No.: 237 003) levels for kidney function tests, were tested using commercial kits (Spectrum Chemical Company, Obour City, Cairo, Egypt). The difference between total protein and albumin was used to compute the globulin level.

#### Cecal microbiota

For microbial analysis, aseptic samples of the cecal content have been collected from every selected bird. The cecal was placed in sterile bags with 50 mL of ice-cold cryoprotective broth^42^, wrapped with plastic, and stored at − 80 °C^43^ until analysis. For all analytical techniques, ceca that had been deep-frozen were removed from the storage bags after being thawed for 20 min. The contents of the cecal digest were then aseptically moved into a new, sterile bag. It had been diluted ten times (10% wt/vol) in sterile, ice-cold anoxic Phosphate-Buffered Saline (PBS 0.1 M, PH 7.0) and homogenized for three minutes. The digesta slurries were then treated in the manner described below. Each cecal digest homogenate was serially diluted (from 10 − 1 to 10 − 7) using PBS (1 mL). *Bacillus SPP, Escherichia coli (E. coli), Clostridium Spp and Enterococcus Spp* were counted using serial dilutions made from around 1 g of cecal digesta that had been collected and homogenized. After plating dilutions, the bacterial target groups were detected on Salmonella shigella agar, MacConkey agar, and duplicate selective agar media M.R.S^44^. Colony-forming units (log10 CFU/g) were measured after plates were cultured for 48 h at 37 °C.

#### Statistical analysis

The statistical analysis was carried out utilizing a completely randomized design and SAS 9.2's general linear models (GLM) technique^45^. Pens served as the experimental unit for all analyses. The data were analyzed using one-way ANOVA. We utilized Duncan multiple range tests to compare means. Significance was defined as *P* < 0.05, and a trend toward significance as 0.05 < *P* < 0.10. *P* values less than 0.001 are denoted as "*P* < 0.001" instead of the real value.

## Results

The weekly temperature-humidity index (THI) is shown in Table 2. Results showed that the birds were exposed to severe heat stress throughout the duration of the experiment, with the mean minimum and maximum THI values of 29.18 and 34.27, respectively.

**Table 2:** Weekly fluctuations in room temperatures, the relative humidity (RH), and the corresponding temperature-humidity index (THI).

(weeks)	Min Temp °C	Max Temp °C	RH %	Min THI	Max THI
1	34.7	39.3	38	30.79	34.51
2	33.3	39.4	36	29.55	34.44
3	32.7	38.9	39	29.24	34.26
4	31.5	38.2	42	28.42	33.92
5	32.5	39.5	32	28.68	34.21
Mean	32.74	39.06	37.4	29.18	34.27

THI = Tdb—[(0.31–0.31 RH)* (Tdb − 14.4)]. THI- Temperature-humidity index. Tdb- Dry bulb temperature in Celsius. RH—Relative humidity.

### Growth performance

There was no mortality during the experiment periods. Table 3 shows the effects of dietary inclusion of propolis (PRO), *Spirulina platensis* (SP), and their mixture on the growth performance of Japanese quail chicks. When compared to the control, SP and MIX groups, dietary including PRO at a concentration of 400 mg/kg significantly (*p* < 0.01) increased body weight (BW) at 35 and 40 days of age, and improved (*p* < 0.01) body weight gain (BWG) of quails over the periods 29–35 and 7–42 days of age, respectively. However, feed intake (FI) was unaffected. When compared to the control group, the dietary inclusion of MIX dramatically decreased feed consumption and BWG while also improving FCR during the period from 7 to 42 days of age (*p* < 0.01). Supplementing SP at a dose of 1 g/kg significantly reduced FI and improved BWG and FCR of quails compared to the control and PRO groups between 29–35 and 7–42 days of age (*p* < 0.01). supplementation of mixture of SP and PRO significantly (*p* < 0.01) decreased BW at 28 and 35 days of age, as well as BWG and FI of quails between 22–28 and 7–42 days of age, respectively. However, FCR improved between 22–28 and 7–42 days of age compared to the control group.

**Table 3:** Effects of dietary inclusion of propolis (Pro), *Spirulina platensis* (SP), and their mixture on productive performance of Japanese quail chicks.

Items	Treatments (Mean ± SD)				SEM	*P* value
	Control	Pro	SP	Mix		
Body Weight, g/bird						
IW (7 days)	26.32	27.05	25.80	26.69	0.217	0.212
14 days	55.92	56.67	54.55	55.97	0.526	0.573
21 days	102.77	105.82	105.02	102.11	0.671	0.152
28 days	152.52^a^	153.59^a^	151.15^a^	146.62^b^	0.858	0.011
35 days	194.61^b^	201.49^a^	195.00^b^	190.48^c^	1.008	0.001
42 days	220.27^**b**^	228.64^a^	219.51^b^	217.15^b^	1.082	0.001
Body weight gain, g/bird						
7–14 days	29.61	29.62	28.75	29.29	0.374	0.849
15–21 days	46.85^b^	49.148^a^	50.46^a^	46.13^b^	0.489	0.001
22–28 days	49.74^a^	47.77^ab^	46.13^bc^	44.51^c^	0.592	0.004
29–35 days	42.09^c^	47.89^a^	43.86^ab^	43.86^ab^	0.778	0.044
36–42 days	26.16^a^	27.15^a^	24.51^b^	26.67^a^	0.296	0.003
7–42 days	194.45^b^	201.58^a^	193.71^bc^	190.47^c^	0.999	0.001
Feed intake, g/ bird						
7–14 days	88.16	88.33	89.00	85.67	0.524	0.112
15–21 days	134.28^c^	142.75^ab^	148.00^a^	139.37^bc^	1.498	0.003
22–28 days	149.48^a^	146.18^a^	149.61^a^	133.35^b^	1.600	0.001
29–35 days	152.81^a^	149.72^a^	130.44^b^	133.80^b^	2.361	0.001
36–42 days	113.75^a^	113.70^a^	105.27^b^	112.01^a^	1.019	0.002
7–42 days	638.99^a^	637.06^a^	619.22^b^	604.16^c^	3.302	0.001
Feed conversion ratio						
7–14 days	2.987	2.985	3.110	2.929	0.033	0.298
15–21 days	2.865	2.906	2.938	3.021	0.025	0.155
22–28 days	3.005	3.061	3.249	3.013	0.039	0.090
29–35 days	3.644^a^	3.157^b^	2.976^b^	3.051^b^	0.071	0.001
36–42 days	4.352	4.190	4.303	4.205	0.043	0.521
7–42 days	3.286^a^	3.160^b^	3.196^b^	3.173^b^	0.014	0.002

^a,b,c^ Means within the same row carrying different superscripts are significantly different at (*P* < 0.05). Control: control, Pro: basal diets supplemented with propolis at 400 mg/kg, SP: basal diets supplemented with *Spirulina platensis* powder at 1g/kg, Mix: basal diets supplemented with propolis at 400 mg/kg + *Spirulina platensis* powder at 1g/kg.

### Nutrient digestibility

The effects of adding propolis (PRO), *Spirulina platensis* (SP), and their combination to the diet on nutrient digestibility of Japanese quail chicks are displayed in Table 4**.** Supplementation of propolis at a level of 400 mg/kg to quails diet significantly improved ether extract (*P* < 0.001) and crude protein (*P* < 0.028) digestibility when compared to the control and SP and MIX groups. When compared to the control and PRO groups, dietary including SP at a concentration of 1 g/kg alone or in combination with 400 mg Pro/kg significantly (*p* < 0.001) reduced nutrient digestibility of ether extract. However, dry matter digestibility was not affected by treatments.

**Table 4:** Effects of dietary inclusion of propolis (Pro), *Spirulina platensis* (SP), and their mixture on nutrient digestibility of Japanese quail chicks.

Items	Treatment				SEM	*P* value
	Control	Pro	SP	MIX		
DM	68.95	70.76	66.87	67.15	0.680	0.149
EE	76.15^b^	79.89^a^	69.48^c^	71.00^c^	1.011	0.001
CP	69.01^b^	73.39^a^	68.12^b^	68.01^b^	0.769	0.028

^a,b,c^ Means within the same row carrying different superscripts are significantly different at (P < 0.05). DM: Dry matter. EE: Ether extract. CP: Crude protein. Control: control, PRO: basal diets supplemented with propolis at 400 mg/kg, SP: basal diets supplemented with *Spirulina platensis* powder at 1g/kg, Mix: basal diets supplemented with propolis at 400 mg/kg + *Spirulina platensis* powder at 1g/kg.

### Carcass characteristics

The effects of adding propolis (PRO), *Spirulina platensis* (SP), and their combination to the diet on the carcass characteristics of Japanese quail chicks are displayed in Table 5. In comparison to the control group, supplementing SP at a dose of 1 g/kg either alone or in combination with 400 mg Pro/kg greatly enhanced (*P* < 0.011) the gizzard weight percentage. In comparison to the SP and Mix groups, liver weight was dramatically (*P* < 0.046) decreased by a diet that included 400 mg/kg of propolis. However, dressing, heart, small intestine weights, and cecum length were unaffected by dietary factors (Pro, SP, and Mix).

**Table 5:** Effects of dietary inclusion of propolis (Pro), *Spirulina platensis* (SP), and their mixture on carcass traits of Japanese quail chicks.

Items	Control	Treatment			SEM	*P* value
		Pro	SP	MIX		
LBW g	194.02	194.73	192.95	180.00	5.489	0.777
Dressing%	73.082	70.354	75.165	74.735	0.681	0.056
Liver%	2.119^ab^	1.804^b^	2.233^a^	2.301^a^	0.066	0.046
Gizzard%	1.289^c^	1.382^cb^	1.478^ab^	1.579^a^	0.035	0.011
Heart%	0.841	0.874	0.806	0.839	0.014	0.429
Small intestine W%	2.384	1.964	2.653	2.361	0.104	0.150
Cecum W %	0.680	0.528	0.972	0.848	0.065	0.078
Cecum L (cm)	7.4	7.5	8	7.3	0.195	0.540

^a,b,c^ Means within the same row carrying different superscripts are significantly different at (*P* < 0.05). LBW: Live body weight. Control: control, PRO: basal diets supplemented with propolis at 400 mg/kg, SP: basal diets supplemented with *Spirulina platensis* powder at 1g/kg, Mix: basal diets supplemented with propolis at 400 mg/kg + *Spirulina platensis* powder at 1g/kg.

### Blood biochemistry

#### Kidney function

**Fig. 1:**
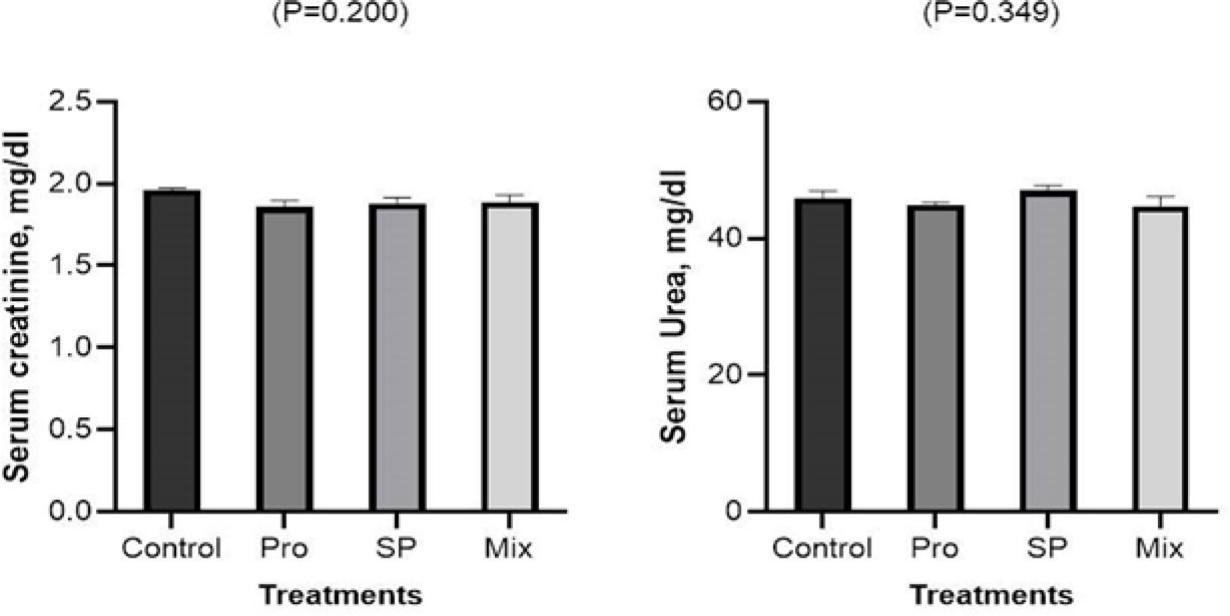
Effects of dietary inclusion of propolis (PRO), *Spirulina platensis* (SP), and their mixture on kidney function of Japanese quail chicks.

The findings of the kidney function as affected by feeding of Pro, SP, and their combination to Japanese quail chicks are displayed in (Fig. 1). The serum concentrations of creatinine and urease in Japanese quail chicks were not affected by the diet, either alone or in combination with Pro and SP.

#### Total protein and albumin

**Fig. 2:**
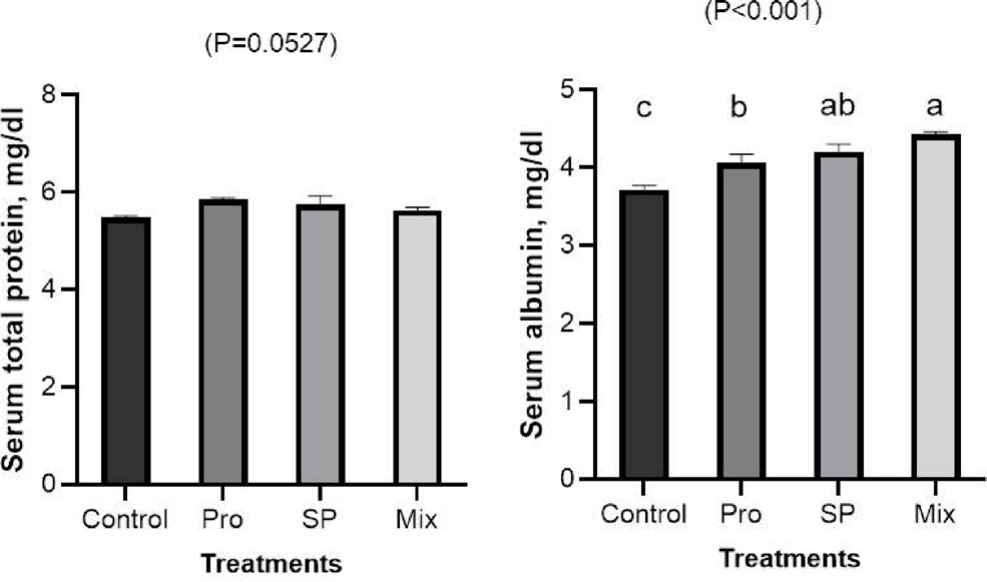
Effects of dietary inclusion of propolis (PRO), *Spirulina platensis* (SP), and their mixture on serum concentration of total protein and albumin of Japanese quail chicks. Bars with different letters (**a**,**b**,**c**) are significantly different (*p* < 0.05).

The results of measuring the serum concentrations of total protein and albumin after feeding Japanese quail chicks Pro, SP, and their combination are displayed in (Fig. 2). In comparison to the control group, the addition of 400 mg Pro/kg or 1g SP/kg, either alone or in combination, dramatically (*P* < 0.01) increased the albumin concentration in the serum. The Mix group had the highest amount of albumin found. The treatments, however, had no effect on the serum total protein concentration.

#### Total cholesterol and triglyceride

**Fig. 3:**
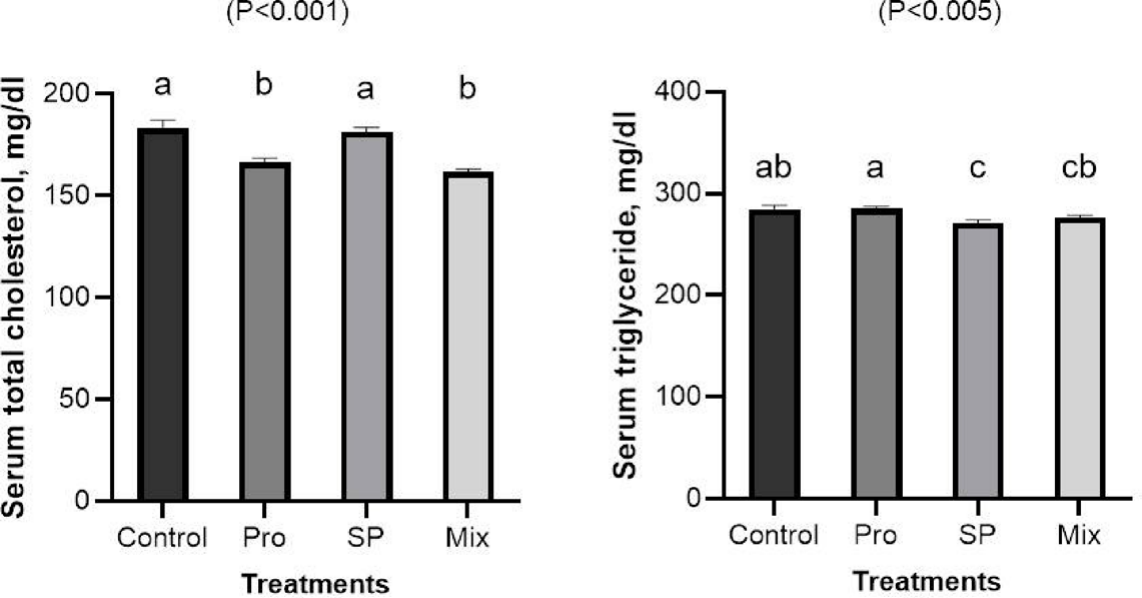
Effects of dietary inclusion of propolis (Pro), *Spirulina platensis* (SP), and their mixture on serum concentration of total cholesterol and triglyceride of Japanese quail chicks. Bars with different letters (**a**,**b**,**c**) are significantly different (*p* < 0.05).

Figure 3, shows the results of measuring the serum concentrations of triglyceride and total cholesterol after Japanese quail chicks were fed Pro, SP, and their combination under chronic heat-stressed. The addition of 1g SP/kg, either by alone or in combination with 400 mg Pro/kg, significantly decreased the serum levels of triglycerides as compared to the control or Pro groups. Furthermore, group Pro and Mix had lower (*P* < 0.001) serum concentrations of total cholesterol than the control and SP groups of Japanese quail chicks.

#### Glucose

**Fig. 4:**
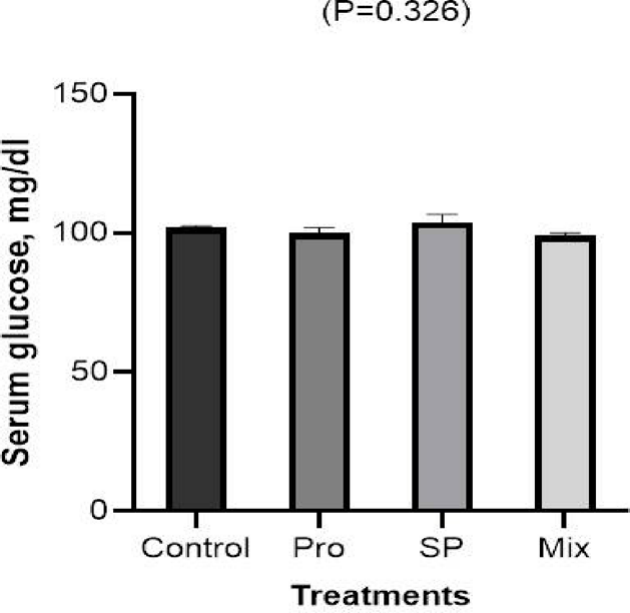
Effects of dietary inclusion of propolis (PRO), *Spirulina platensis* (SP), and their mixture on serum concentration of glucose of Japanese quail chicks.

After Japanese quail chicks were given Pro, SP, and their combination, (Fig. 4) displays the results of analyzing the serum concentrations of glucose under chronic heat-stressed. Treatments had no effect on the serum glucose level.

### Cecal microbiota

The effects of adding propolis (PRO), *Spirulina platensis* (SP), and their combination to the diet on cecal microbiota of Japanese quail chicks are displayed in Table 6. When quails were fed a diet supplemented with 400 mg/kg of propolis, their counts of *Bacillus SPP*, *Escherichia coli (E. coli), Clostridium Spp, and Enterococcus Spp* were significantly lower than those of the control and SP groups (*P* < 0.001). *E. Coli* counts was considerably (*p* < 0.01) lower in the dietary group that included SP at a concentration of 1 g/kg than the control group. In comparison to the control and SP groups, quails fed a diet supplemented with a mixture of propolis and *Spirulina platensis* powder had considerably lower levels of *Clostridium Spp* and *Enterococcus Spp.*

**Table 6:** Effect of propolis (Pro), *Spirulina platensis* (SP), and their mixture on cecal microbiota of Japanese quail chickens.

Items	Treatment				SEM	*P* value
	Control	Pro	SP	MIX		
*Bacillus SPP*	6.07^a^	5.60^b^	6.29^a^	6.00^a^	0.083	0.001
*Clostridium SPP*	5.02^b^	4.50^c^	6.40^a^	4.11^d^	0.225	0.001
*E. Coli*	7.30^a^	3.01^c^	5.01^b^	7.20^a^	0.457	0.001
*Enterococcus SPP*	5.43^b^	3.17^d^	6.93^a^	4.51^c^	0.354	0.001

^a,b,c^ Means within the same row carrying different superscripts are significantly different at (*P* < 0.05). Control: control, PRO: basal diets supplemented with propolis at 400 mg/kg, SP: basal diets supplemented with *Spirulina platensis* powder at 1g/kg, Mix: basal diets supplemented with propolis at 400 mg/kg + *Spirulina platensis* powder at 1g/kg.

## Discussion

One significant environmental component that negatively affects animals’ immunity and general health is heat stress. Improving the health and productive of broilers raised in hot climates is crucial to maintaining their output. Previous research suggests that adding propolis to animal species diets can enhance taste^46^. The high phenolic acids and flavonoids concentrations of PRO could enhance performance by promoting healthy gut microbiota, lowering pathogenic bacterial counts, establishing a stable intestinal flora, and improving digestion. In the current study, Dietary including propolis at 400 mg/kg significantly improved body weight, and body weight gain in quails aged 29–35 and 7–42 days, compared to the control, SP, and MIX groups. When compared to the control group, the dietary inclusion of MIX dramatically decreased feed consumption and BWG while also improving FCR during the period from 7 to 42 days of age. Bee propolis was improved a variety of productive characteristics in livestock animals, including immunity, body weight and feed efficiency^47,48^. The current findings agree with those of Dosoky et al.^49^ who indicated that dietary including PRO 100, 200 and 300 mg/kg significantly increased body weight of broilers at 21 days of age compared to the control group. Furthermore, broiler fed a diet supplemented with 1000 mg propolis/kg during 22–42 days of age showed a substantial improvement in BW, BWG, and FI when compared to the control group^50^. According to Fouad et al.^51^ Japanese quail fed diet supplemented with PRO at 200, 400, and 600 mg/kg showed a substantial (*P* < 0.05) improvement in FI, BW, BWG, and FCR when compared to the control group. Hassan et al.^47^ found a significant increase in body weight of broilers fed diet added with propolis at 1, 2 and 3 g/kg compared to the control group. However, compared to the control broiler groups, Attia et al.^52^ observed a decrease (*P* < 0.05) in FI in the broiler groups treated with 300 mg/kg of propolis from 0 to 35 days of age. Attia et al.^52^ came to the conclusion that decreased FI in PRO-treated groups had no effect on the physiological growth of the gastrointestinal system based on the relative weights of organs (gizzard, heart, liver, pancreas, intestine, and abdominal fat) determined in their study. The variations in propolis supplementation dosages could be the cause of the disparities in results. In the present study, supplementing SP at a dose of 1 g/kg significantly reduced FI and improved BWG of quails compared to the control and PRO groups between 29–35 and 7–42 days of age (*p* < 0.01). The primary outcome of improving FCR is a decrease in FI. The feed conversion ratio and body weight gain were positively impacted by the addition of spirulina^53–55^. Alwaleed et al.^56^ found similar results, showing that broilers given 1% SP had a significantly improvement of FCR than the control group. Spirulina has a considerable favorable impact on growth performance metrics due to its high nutritional profile, which includes all the necessary amino acids, vitamin C, vitamin B Complex, mineral elements, antioxidant carotenoids, and important fatty acids. Furthermore, by promoting the synthesis of Ig A and Ig E and fostering a healthy microbiota, spirulina improves mucosal immunity^30,57^. In quail chicks, ovo injection of SP improved the expression of genes linked to immunity, antioxidants, and hatchability^58^. According to Ibrahim et al.^59^ indicated that dietary including 0.5, 1, and 2 g/liter of spirulina to drinking water for four weeks significantly improved health, the average body weight gain, feed conversion ratio (FCR), feed efficiency, and European Production Efficiency values. Although results are susceptible to variation based on the species and environmental conditions of examined. Certain bioactive compounds in Propolis and Spirulina may interact in a way that does not enhance—and may even slightly inhibit each other individual effects.

Propolis may indirectly increase the digestion of nutrients, according to some theories^60^. Propolis’ ability to modify gut bacteria by favoring beneficial bacteria and inhibiting harmful ones is responsible for this impact^61^. A healthy bacterial structure can support the immune system and the release of digestive enzymes, which enhances intestinal nutrition absorption and digestion^62^. In the current finding, under heat stress conditions supplementation of propolis at a level of 400 mg/kg to broilers diet significantly improved ether extract (*P* < 0.001) and crude protein digestibility (*P* < 0.028) when compared to the control and SP and MIX groups. This improvement could be due to improved intestinal architecture and nutrient absorption, as well as increased activity of the enzymes amylase, saccharase, and phosphatase^63,64^. In the current study, dietary including SP at a concentration of 1 g/kg alone or in combination with 400 mg Pro/kg significantly (*p* < 0.001) reduced nutrient digestibility of ether extract. *Spirulina platensis* contains a relatively high amount of crude fiber and ash, which can interfere with fat digestion and absorption in the gastrointestinal tract. The presence of indigestible components such as cell wall polysaccharides may encapsulate lipids, making them less accessible to digestive enzymes and thus reducing ether extract digestibility. Additionally, the increased viscosity of digesta due to microalgae inclusion can hinder the emulsification and absorption of dietary fats, further contributing to lower digestibility values. These factors collectively suggest that the structural and compositional characteristics of Spirulina can negatively impact the efficiency of fat utilization in broilers, necessitating further research to optimize its use in poultry diets^65^.

In the current study, supplementing SP at a dose of 1 g/kg either alone or in combination with 400 mg Pro/kg greatly enhanced (*P* < 0.011) the relative gizzard weight. In comparison to the SP and Mix groups, liver weight was dramatically (*P* < 0.046) decreased by a diet that included 400 mg/kg of propolis. Similar outcomes were attained by, Mahmoud et al.^66^ the inclusion of propolis at doses of 100, 250, 500, and 750 mg in broiler diets resulted in reduced weights of the carcass, liver, heart, and gizzard. However, Fouad et al.^51^ indicated that Japanese quail fed diet supplemented with PRO at 200, 400, and 600 mg/kg showed a substantial (*P* < 0.01) increased in relative weight of gizzard, heart, liver, spleen, and intestine when compared to the control group. The advantages of employing such dietary spirulina supplementation were further supported by Ribeiro et al.^67^ who demonstrated an increase in structural muscle protein synthesis in piglets fed diets containing lysozyme and spirulina.

Various physiological changes in animals, such as those related to age, species, nutrition, season, and physiological state, can be reflected in blood biochemical profiles^68^. In the current study, the addition of 400 mg Pro/kg or 1g SP/kg, either alone or in combination, dramatically (*P* < 0.01) increased the albumin concentration in the serum. The Mix group had the highest amount of serum albumin compared to the control group. In the current study, under chronic heat-stressed addition of 1g SP/kg, either by alone or in conjunction with 400 mg Pro/kg, significantly decreased the serum levels of triglycerides as compared to the control or Pro groups. Similar outcomes were attained by, Moustafa et al.^53^ who indicated that dietary including SP at 0.5, 1 and 1.5% significantly reduced serum serum triglyceride concentration. After 14 days of prolonged heat stress, broiler concentrations of plasma triglycerides and liver enzymes dramatically increased^69^. Broilers exposed to heat stress showed a decrease in blood cholesterol and triglycerides when supplemented with 0.5 and 2% *spirulina*^70,71^. In the present study, dietary including PRO at 400 mg/kg alone or in combination with SP had lower (*P* < 0.001) serum concentrations of total cholesterol than the control and SP groups of Japanese quail chicks.

Poultry’s immune system, digestion, and general health are all significantly impacted by their gut microbiota. Because of its antibacterial, antioxidant, and immunomodulatory qualities, propolis a natural feed additive has drawn interest as an antibiotic substitute for gut microbiota regulation^51^. In this research, When quails were fed a diet supplemented with 400 mg/kg of propolis, their counts of *Bacillus SPP, E. coli, Clostridium Spp,* and *Enterococcus Spp* were significantly lower than those of the control and SP groups (*P* < 0.001). A reduction in these bacterial populations raises the possibility that propolis enhances intestinal integrity and avian health in general. Propolis may reduce the hypothalamic–pituitary–adrenal (HPA) axis reaction brought through heat stress by enhancing lactobacilli and bifidobacteria and decreasing total aerobics and coliform bacteria in broiler chicks’ guts^72^. Bioactive substances including terpenes, phenolic acids, and flavonoids, which are abundant in propolis, have been demonstrated to have broad-spectrum antibacterial effects by rupturing bacterial cell walls and reducing the activity of bacterial enzymes^73^. In contrast, phycocyanin, polysaccharides, and important fatty acids like gamma-linolenic acid are rich in spirulina^74^. By influencing immunological responses and functioning as fermentable substrates, these substances have been demonstrated to promote the growth of advantageous bacteria such as *Lactobacillus*^75^. Spirulina-derived polysaccharides, in particular, may influence microbial fermentation patterns, increasing the production of short-chain fatty acids (SCFAs), which are crucial for maintaining intestinal health and suppressing pathogenic population. Whereas the MIX group did not show superior outcomes in all the parameters of interest compared to the single-supplement groups, it did show great improvements in some areas such as lipid metabolism and regulation of gut microbiota. In the current study, *E. Coli* counts was considerably (*p* < 0.01) lower in the dietary group that included SP at a concentration of 1 g/kg than the control group. The effects of SP supplementation on gut microbiota in laying quails under heat stress have been the subject of numerous investigations; varying doses of SP supplementation have reduced the *E. coli* count in the ileal content without affecting the lactobacillus count at day 134^76^. According to Park et al.^77^ supplementing with spirulina raised the concentration of *Lactobacillus* in the cecum, but had no effect on the quantity of coliform bacteria. It was discovered that broilers given feed supplemented with 0.5, 1, and 1.5% had considerably lower intestinal E. coli and Salmonella count values (*P* < 0.05) than control^78^. Quails fed a diet supplemented with a mixture of propolis and *Spirulina platensis* powder had significantly lower levels of *Clostridium Spp* and *Enterococcus Spp* In comparison to the control and SP groups*.* This suggests a potential complementary or additive antimicrobial interaction between propolis and spirulina. The reduction of *Clostridium* spp. and *Enterococcus* spp., both of which can contribute to gastrointestinal disturbances and opportunistic infections, implies an improvement in gut microbial balance. The combination treatment may thus enhance gut health, reduce pathogenic colonization, and possibly support the host’s immune response. Further research is needed to explore the mechanisms behind its antimicrobial effects and its impact on gut microbiota composition at the molecular level.

## Conclusion

Overall, the present results indicated that propolis at 400 mg/kg and *Spirulina platensis* powder at 1 g/kg, when used individually as feed additives, improved the performance and health status of Japanese quail chicks during the period from 7 to 42 days of age and enhanced nutrient digestibility under heat stress conditions. Both additives showed beneficial effects across various parameters. While propolis supplementation was associated with notable improvements in growth performance, nutrient digestibility and reducing the level of harmful bacteria, *Spirulina platensis* also demonstrated positive effects, particularly in feed conversion ratio (FCR), reducing the level of *E.Coli*, serum albumin, and triglyceride levels. The combination of both additives did not outperform individual supplementation. Future studies could explore different dosage levels and investigate their long-term effects on poultry health and productivity.

## Data Availability

The datasets generated and/or analyzed during the current study are available from the corresponding author on reasonable request.

## Abbreviations

AOAC: Association of official analytical chemists
BW: Body weight
BWG: Body weight gain
CP: Crude protein
DM: Dry matter
*E. Coli*: *Escherichia coli*
EE: Ether extract
FCR: Feed conversion ratio
FI: Feed intake
PRO: Propolis
RH: Relative humidity
SP: *Spirulina platensis*
Tdb: Dry bulb temperature in celsius
THI: Temperature-humidity index
